# Obtaining and Characterization of an Interspecific Hybrid between *Lilium callosum* and ‘Snow Queen’ and Evaluation of the *Botrytis* Stress Response

**DOI:** 10.3390/plants13101376

**Published:** 2024-05-15

**Authors:** Yongyao Fu, Shulin Lu, Chengchen Liu, Chaojun Ding, Xiaoyu Wang, Xinrong Li, Sijia Jiang, Liping Yang

**Affiliations:** 1 School of Advanced Agriculture and Bioengineering, Yangtze Normal University, Chongqing 408100, China; 2 Heilongjiang Forest Botanical Garden, Harbin 150046, China

**Keywords:** *Lilium*, distant hybridization, morphology, SSR marker, *Botrytis* resistance

## Abstract

To cultivate excellent lily germplasms, an interspecific hybrid (LC×SQ-01) was successfully obtained by using a cut-style pollination method in which the rare wild lily *Lilium callosum* was used as the female parent and the cut flower *L. longiflorum* ‘Snow Queen’ was used as the male parent. The morphological features of LC×SQ-01 included height, leaf length, and width, which were observed to be between those of the parents in the tissue-cultured seedlings. The height and leaf length of LC×SQ-01 were more similar to those of the male parent, and the width was between the widths of the parents for field-generated plants. The epidermal cell length and the guard cell and stoma sizes were between those of both parents in tissue-cultured and field-generated plants. In addition, the shapes of the epidermal cells and anticlinal wall in LC×SQ-01 were more analogous to those in the male parent, while the stoma morphology was different from that of both parents. Fourteen pairs of polymorphic SSR primers were identified in both parents, and the validity of LC×SQ-01 was demonstrated by PCR amplification using five pairs of SSR primers. Flow cytometry and root tip squashing assays revealed that LC×SQ-01 was a diploid plant, similar to its parents. Furthermore, the LC×SQ-01 hybrid was more resistant to *B. cinerea* than its parents, and it also showed much greater peroxidase (POD) and catalase (CAT) activity than the parents. These results lay a foundation for breeding a new high-resistance and ornamental lily variety.

## 1. Introduction

*Lilium* is a world-renowned fresh-cut flower that occupies a relatively large share of Chinese and international markets. China is the natural distribution centre of lily, with approximately 55 native wild lily species, accounting for half of the world’s wild lily resources. The abundance of lily species in China provides a good foundation for the breeding of new lily varieties with independent intellectual property rights [1]. The intraspecific and interspecific hybridization of lily is an important means of breeding new lily varieties that can freely combine excellent genetic traits. Currently, there are some reports on the breeding of hybrid lilies. Huang et al. (1985) [2] used *L. longiflorum* as the female parent and *L. davidii* var. *unicolour* as the male parent to obtain a hybrid, *Lilium longi-davidii* (‘She Lan’), with a light aroma, red flesh, and separate petals. The hybrid lines ‘Gelria’ and ‘Flevo’ of *L. longiflorum* were used as the female parents, and *L. dauricum* was used as the male parent to obtain hybrid offspring resistant to *Fusarium* [3]. The Asiatic lily ‘Brunello’ was crossed with the wild lily *L. pumilum* to breed the new food and ornamental variety ‘Dandie’ [4]. Masumi Yamagishi (2023) [5] successfully introduced a floral fragrance trait by crossing Asiatic lily with *L. cernuum*. However, at present, the wild resources used in lily breeding are still very limited, and many original lily species with unique and excellent traits remain to be further developed and utilized.

*L. callosum* is a rare lily resource native to northeastern China with red or light red flowers, drooping recurved tepals, and strong cold and salinity tolerance and is an excellent material for resistance breeding [6,7]. ‘Snow Queen’ is a hybrid line of *L. longiflorum* with white flowers, a trumpet shape, a strong fragrance, a short growth period, and strong heat tolerance and is an excellent lily variety for cut flowers, but there is an urgent need to improve its resistance to grey mould, which is caused by *Botrytis elliptica* or *B. cinerea* in lilies [8,9,10]. *L. callosum* and *L. longiflorum* hybrid ‘Snow Queen’ both belong to the diploid plants (2n = 2x = 24). And, ‘Snow Queen’ can be used as a mother or father parent for hybridization [11,12]. In the International Lily Register and Checklist (2007) Fifth Supplement [13], *L. callosum* belongs to Division I Asiatic hybrids, that are derived from the intraspecific hybridization of the Sinomartagon family (including *L. auricum*, *L. lancifolium*, etc.) [14], while ‘Snow Queen’ belongs to Division V *longiflorum* lilies that are formed through the intraspecific hybridization of the Leucolirion line [15]. Currently, there are no reports on the hybrid breeding of the wild *L. callosum* and the cut flower ‘Snow Queen’. Therefore, the cultivation of an interspecific hybrid between *L. callosum* and ‘Snow Queen’ could be very meaningful today.

The identification of lily hybrids mainly includes morphological observations [11,16] and molecular marker identifications [17,18]. Among these approaches, morphological observations are convenient and practical. The leaf epidermal characteristics can usually be used to preliminarily determine the genetic relationship between parents and offspring and have been widely applied for this purpose [19,20]. Simple sequence repeat (SSR) molecular markers are tandemly repeated DNA sequences consisting of 1–6 nucleotides that have the advantages of high information content, codominance, and high species specificity and have been successfully applied in the identification of lily hybrids [21,22].

Cut-style pollination, or intrastylar pollination, can prevent the pre-fertilization obstacle caused by the stigma or style and overcome the incompatibility of distant crosses [23,24]. In our previous studies, a number of different combinations of lily hybrid offspring were obtained by cut-style pollination [7]. To cultivate excellent lily germplasms using a wild resource and a poplar cut flower, in this study, *L. callosum* was chosen as the female parent, ‘Snow Queen’ was chosen as the male parent, cut-style pollination was performed for hybridization, and F1 hybrids were obtained. Early morphological observations in one of the hybrids were carried out using both tissue-cultured and open-field seedlings, the authenticity and chromosome ploidy of the hybrids were identified by SSR molecular markers and flow cytometry, and the antioxidant enzyme activities and *Botrytis* stress response were analysed to provide scientific evidence for the future cultivation of new lily germplasms with excellent flower colours and high resistance.

## 2. Materials and Methods

### 2.1. Plant Materials

*Lilium callosum* Siebold & Zucc. and ‘Snow Queen’, a *L. longiflorum* hybrid line, were cultivated at the experimental base of the Flower Genetic Breeding Team of Yangtze Normal University.

### 2.2. Cross-Pollination and Seed Culture

Triphenyltetrazolium chloride (TTC) staining and liquid culture methods were used to detect the pollen fertility and viability of ‘Snow Queen’, respectively [25]. The *L. callosum* stigmas were collected in the flowering stage, and the receptivity was observed by putting the stigmas into a benzidine hydrogen peroxide reaction solution (1% benzidine: 3% hydrogen peroxide: water = 4:11:22, volume ratio). *L. callosum* plants with good growth were emasculated and bagged one day before flowering. The cut-style pollination method was used for cross-pollination on the day of flowering, and a total of 68 flowers were pollinated. Immediately after pollination, the pistils were wrapped in aluminium foil, which was removed after one week, after which the growth and swelling of the ovaries were observed.

At 20 days after pollination, the enlarged ovaries of 35 flowers were cut for primary culture. The ovaries were sliced into 0.3 cm thick sections and cultured in ovary culture medium (MS + NAA 0.1 mg L^−1^) for approximately 30 days. The enlarged ovules were peeled and cultured on MS medium. After observing the fruit swelling in another 33 flowers, the seeds with embryos were selected and inoculated in MS + 6-BA 0.5 mg L^−1^ + NAA 0.2 mg L^−1^ solid medium. The culture temperature in the tissue culture room was 22 °C, the light intensity was approximately 5000 lx, and the light duration was 14 h/d.

### 2.3. Morphological Comparison of the Hybrids and Parents

Seedlings grown from hybrid embryos LC×SQ-01 were selected for the multiplication culture, and the parents *L. callosum* and ‘Snow Queen’ were simultaneously subcultured to obtain sterile seedlings. After 2 months, morphological differences between the LC×SQ-01 seedlings and the parents were observed and compared, including differences in leaf shape (leaf length and leaf width), rooting (root length), and leaf epidermal (upper and lower epidermal cells and stomata) characteristics, based on the previous description [16]. Briefly, 10 leaf upper or lower epidermis from at least 3 leaf samples of the LC×SQ-01 were separated by hand, and 50 versions were observed under an optical microscope (Olympus BX53, Tokyo, Japan). The same operation was performed in the parents *L. callosum* and ‘Snow Queen’, respectively. Moreover, the parents *L. callosum* and ‘Snow Queen’ and the hybrid seedlings LC×SQ-01 were transplanted into the culture room, where they remained for another 2 months. Morphological indicators were observed in the same way, and similar light intensities and light cycles were applied.

### 2.4. Identification of Hybrids with SSR Molecular Markers

Total DNA was extracted using the modified cetyltrimethylammonium bromide (CTAB) method. After electrophoresis through 1% agarose gels, the DNA concentration and purity were determined by differential spectrophotometry, and the total DNA was stored at −20 °C for later use. The 98 pairs of SSR primer sequences used were provided by Prof. Ming Jun and Dr. Leifeng Xu from the Institute of Vegetables and Flowers, the Chinese Academy of Agricultural Sciences (Table S1). The DNAs of *L. callosum* (female parent) and ‘Snow Queen’ (male parent) were used as templates for amplification. According to the polymorphism and clarity of the amplified bands in the parents, the amplification primers were selected and screened to identify the hybrids of *L. callosum* and ‘Snow Queen’.

Amplification by polymerase chain reaction (PCR) was performed using an Applied Biosystems VeritiPro PCR system. The volume of the reaction system was 20 μL: 10 μL of 2× Taq Master Mix, 1.0 μL of template DNA, 0.8 μL of primers, and 8.2 μL of deionized (DI) water. The reaction conditions were as follows: predenaturation at 95 °C for 5 min; 38 cycles of denaturation at 95 °C for 30 s, annealing for 30 s (temperature was determined by the optimal annealing temperature of the primer) and an extension at 72 °C for 30 s; and an extension at 72 °C for 7 min. For the amplification products, electrophoresis through 1.5% agarose gels was performed at a voltage of 80 V for approximately 60 min.

### 2.5. Ploidy Level Identification of Hybrids

Fresh leaves from the tissue-cultured seedlings of the hybrids and parents after approximately 60 days of culture were used as materials, and the ploidy level was analysed using flow cytometry [26]. Fresh young leaves (0.5 cm^2^) were added to a Petri dish, 1 mL of nucleus extract was added dropwise, and the leaves were minced individually with a thin sharp blade until they became a paste. The mixture was filtered through a filtration membrane (400-mesh size) into a 1.5 mL centrifuge tube, and the filtrate (approximately 1.0 mL) was placed in a refrigerator at 4 °C for 5 min. Then, 50 μL of precooled propidium iodide (PI) was added to 0.5 mL of extract, staining was performed at 4 °C in the dark for 30 min, and the sample was transferred to a loading tube for detection. A chromosome ploidy chart was obtained to determine the ploidy levels of the hybrids.

The newly grown root tips were collected from the seedlings of LC×SQ-01 and the parents, and the root tips were soaked in 0.1% colchicine for 24 h and then transferred to Carnoy’s solution (3:1 ethanol/glacial acetic acid, *v*/*v*) for 24 h. The root tips were hydrolyzed with 1 mol/L HCl and dissociated for 10 min at 60 °C. After rinsing with distilled water, the root tips were cut approximately 1 mm long and placed on slides for staining with cabernet dye for 15 min. The chromosome number was observed under a microscope (Olympus BX53, Tokyo, Japan).

### 2.6. Detection of Antioxidant Enzyme Activity and Botrytis Cinerea Resistance

Using 60-day-old seedlings, the activity of superoxide dismutase (SOD) was analysed via the nitrogen blue tetrazolium (NBT) method, the activity of peroxidase (POD) was determined via the guaiacol method, and the activity of catalase (CAT) was determined via ultraviolet (UV) spectrophotometry [27,28]. Each experiment was performed in triplicate.

The *Botrytis* stress response of the hybrids was analysed using a spore suspension inoculation method. The *B. cinerea* stain was isolated from the infected lily leaf and identified by Prof. Lifeng Zhai at Yangtze Normal University [9]. *B. cinerea* was grown on a potato dextrose agar (PDA) medium at 28 °C for about 2 weeks. The suspension of *B. cinerea* spores (1 × 10^5^ cfu/mL) was collected with 0.05% Tween 20 solution, the direct titration method (10 μL/plant) was used to inoculate 30 hybrid and 30 parental bulblet scales, respectively, and the pathogenesis of the bulblet scales was investigated at 0 d, 2 d, 4 d, 7 d, and 9 d after inoculation. With reference to the method of Gao et al. (2018) [8], the *Botrytis* resistance of the hybrids was evaluated. Each experiment was performed in triplicate.

Regarding data statistics, the software applications Excel 2013 and SPSS 25 were used for statistical analysis, the results are expressed as the means ± standard errors, and the significance of the differences (*p* < 0.05) was determined using post hoc multiple comparison (Duncan’s method).

## 3. Results

### 3.1. Cross-Pollination and Seed Culture

**Figure 1:**
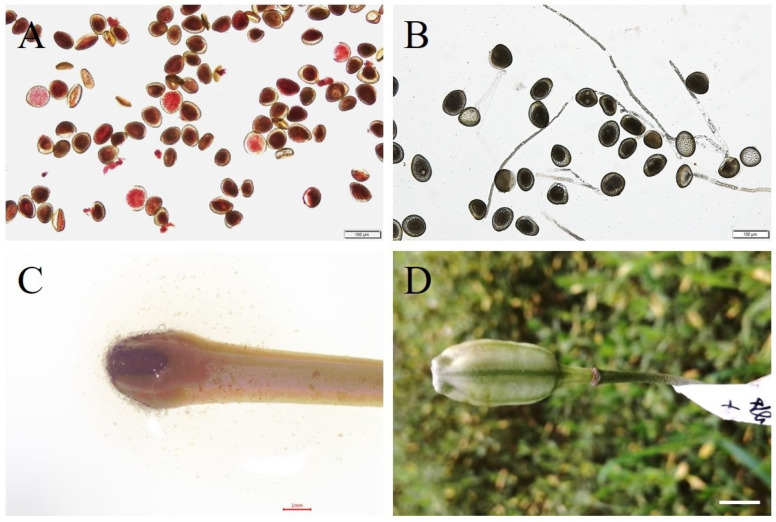
Analysis of pollen vitality of the male parent ‘Snow Queen’ and stigma receptivity in the female parent *L. callosum*. (**A**) Pollen vitality detection using the TTC staining method; (**B**) pollen viability detection using the liquid culture method, bars represent 100 µm; (**C**) stigma receptivity detection using benzidine hydrogen peroxide reaction solution, bars represent 1 mm; (**D**) swollen fruit after 40 days of pollination, bars represent 1 cm.

The percentage of normal fresh pollen in the male parent ‘Snow Queen’ was 82.83%, and the pollen viability measured by TTC staining was 24.35%, while the pollen viability measured by the liquid medium method was 37.4% (Figure 1A,B). Generally, the pollen viability measured by the latter method was more reliable (>30%), ensuring the normal operation of the experiment. At 20 d after cut-style pollination, no swollen ovaries from 35 flowers were found in the embryo rescue culture, and hybrid plants eventually failed to develop (Table 1).

However, in the other group, as shown in Table 1, four fruits from 33 flowers were significantly enlarged at 40 d after the cut-style pollination, as shown in Figure 1C. Sixty days later, the fruit was disinfected, and the seeds with embryos (Table 1, 26 seeds; embryonic rate of 1.4%) were inoculated in MS medium and cultured. After another 40 d, approximately 5 seeds were geminated, but the remaining 21 seeds did not germinate; after 60 d, a total of seven shoots emerged, and the seedlings were obtained at 90 d, which was a rate of 26.9%. In addition, one of the fast-growing seedlings was proliferated and used for further analysis (called LC×SQ-01).

### 3.2. Plant Morphological Characteristics of Hybrid Seedlings

**Figure 2:**
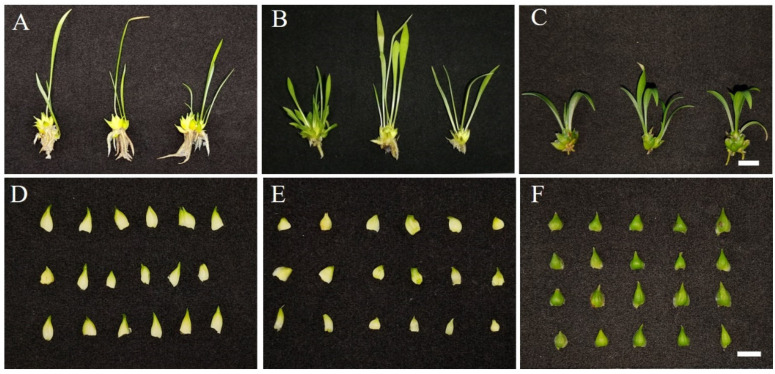
Morphological features of tissue-cultured seedlings of the LC×SQ-01 hybrid and the parents. (**A**–**C**) Plants of *L. callosum*, LC×SQ-01 and ‘Snow Queen’, respectively, cultured for 60 days in medium; the bars represent 1 cm. (**D**–**F**) The bulblet scales from the corresponding seedlings of *L. callosum*, LC×SQ-01 and ‘Snow Queen’, respectively; the bars represent 1 cm.

**Figure 3:**
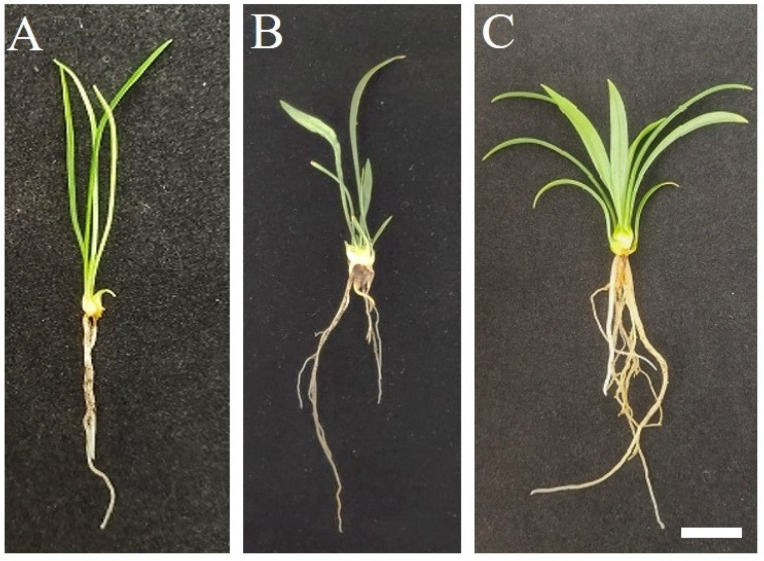
Morphological features of the LC×SQ-01 hybrid and the parents in open-field-grown plants after 60 days. (**A**) *L. callosum*, (**B**) LC×SQ-01, (**C**) ‘Snow Queen’, bars represent 2 cm.

After subculture for 60 days, the average height of 30 hybrid seedlings (LC×SQ-01) was 4.45 cm, the leaf length was 3.71 cm, and the leaf width was 0.16 cm; these values are between those of 30 female parent *L. callosum* seedlings and those of 30 male parent ‘Snow Queen’ seedlings (Table 2). The LC×SQ-01 seedlings had a generally developed root system, which was less developed than that of the female parent but superior to that of the male parent (Figure 2A–C). The morphological characters of the scales of the LC×SQ-01 seedlings were more similar to those of the female parent but different from those in the male parent, which displayed many more dark green colours (Figure 2D–F). The observations of the open-field seedlings cultured for 60 days revealed that the average height of the LC×SQ-01 seedlings was 6.59 cm, the leaf length was 5.08 cm, and the leaf width was 0.27 cm (Table 2). Compared with those of the parents, the height and leaf length were similar to those of the male parent, while the leaf width was between those of the parents. The root growth of the LC×SQ-01 seedlings was greater than that of the female parent and less than that of the male parent, and the thickness of the hybrid seedlings was between those of the parents (Figure 3A–C).

### 3.3. Characteristics of Leaf Epidermal Cells and Stomata in the Hybrid

**Figure 4:**
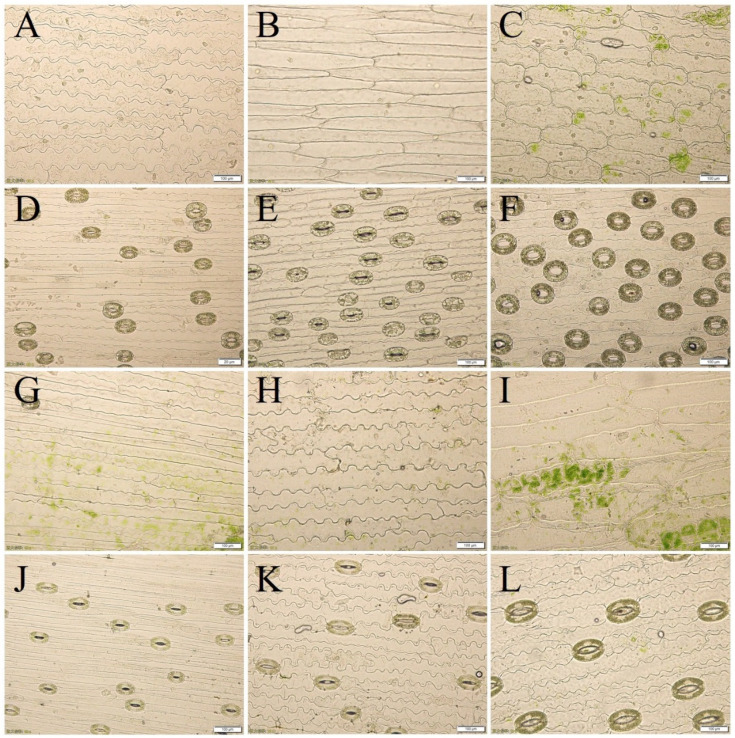
The characteristics of the leaf epidermis and stomata in the LC×SQ-01 hybrid and the parents. (**A**–**C**) Leaf upper epidermis cells of tissue-cultured seedlings in *L. callosum*, LC×SQ-01 and ‘Snow Queen’; (**D**–**F**) leaf lower epidermis cells of tissue-cultured *L. callosum*, LC×SQ-01 and ‘Snow Queen’ seedlings; (**G**–**I**) leaf upper epidermis cells of open-field *L. callosum*, LC×SQ-01 and ‘Snow Queen’ seedlings; (**J**–**L**) leaf lower epidermis cells of open-field *L. callosum*, LC×SQ-01 and ‘Snow Queen’ seedlings. Bars represent 100 μm.

The observations of leaf epidermal cells revealed that the lengths of the upper and lower epidermal cells of both the tissue-cultured and open-field seedlings of the LC×SQ-01 hybrid were between those of the parents, the widths of the upper and lower epidermal cells of the LC×SQ-01 tissue-cultured seedlings were closer to those of the female parent, the widths of the epidermal cells were between those of the parents, and the widths of the lower epidermal cells were the same as those of the parents. The shape of the upper and lower epidermal cells of the LC×SQ-01 tissue-cultured seedlings and the shape of the lower epidermal cells of the open-field seedlings were closer to those of the male parent, while the shape of the upper epidermal cells was irregular, which was different from that of either parent. The shapes of the upper and lower epidermis of the LC×SQ-01 tissue-cultured seedlings were different from those of the parents, i.e., the shape of the lower epidermis was consistent with that of the parents, and the shapes of the upper and lower epidermis of the open-field seedlings were closer to that of the male parent (Table 3, Figure 4).

The genetic relationships and ploidy levels of lily can be preliminarily determined based on stomatal density and size. The observations revealed that the size of the guard cells in the tissue-cultured and open-field seedlings of LC×SQ-01 was between that of the parents, and the shape of the stomatal apparatus was oblong oval, which was different from that of the parents; the stomatal density and stomatal size of the tissue-cultured seedlings in the LC×SQ-01 hybrid were greater than those of the female parent and were similar to those of the male parent (Table 4, Figure 4D–F); and the stomatal size of the open-field seedlings of LC×SQ-01 was between that of the parents, while the stomatal density was closer to that of the female parent (Table 4, Figure 4J–L). A comparison of the open-field and tissue-cultured seedlings revealed that the stomatal density of the male parent decreased the most significantly from 54/mm^2^ to 28/mm^2^; the stomatal density of the LC×SQ-01 hybrid also decreased from 52/mm^2^ to 39/mm^2^, while there was no change in the stomatal density of the female parent.

### 3.4. Identification of Hybrid Using SSR Molecular Markers

**Figure 5:**
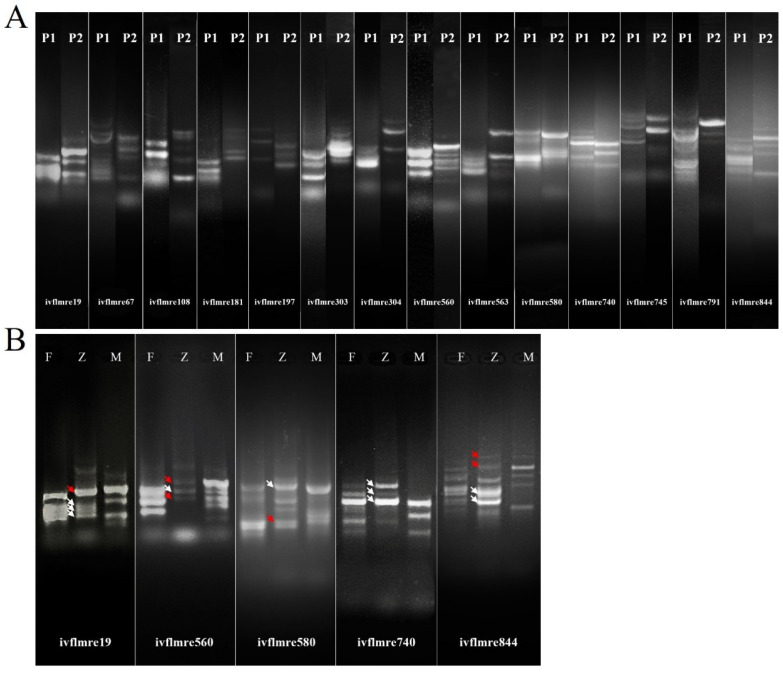
Polymorphism primer screening of the parents and PCR identification of the LC×SQ-01 hybrid. (**A**) P1 is the female parent *L. callosum*, P2 is the male parent ‘Snow Queen’, (**B**) F is the female parent *L. callosum*, Z is the LC×SQ-01 hybrid, and M is the male parent ‘Snow Queen’. The white arrows indicate common bands between the LC×SQ-01 hybrid and female parent *L. callosum*, and the red arrows indicate specific bands between the LC×SQ-01 hybrid and male parent ‘Snow Queen’.

The screening results of 98 pairs of primers showed that clear bands were amplified with 23 pairs of primers for the female parent *L. callosum* and 25 pairs of primers for the male parent ‘Snow Queen’ (Table S2), and finally, polymorphic bands were amplified with 14 pairs of primers for both parents (Figure 5A). Based on the difference in band amplification, five pairs of SSR primers with clear and reproducible band amplification were selected for the identification of hybrids. The amplification results of the five pairs of SSR primers showed that the amplified bands from ivflmre19, ivflmre560, ivflmre580, and ivflmre844 in the hybrid contained both bands specific to the male parent ‘Snow Queen’ and bands identical to those of the female parent *L. callosum*; the exception was Primer ivflmre740, whose amplified bands contained only bands that were identical to those of the female parent (Figure 5B). These results confirmed that the LC×SQ-01 hybrids were real interspecific hybrids.

### 3.5. Analysis of Ploidy Level in the Hybrid

**Figure 6:**
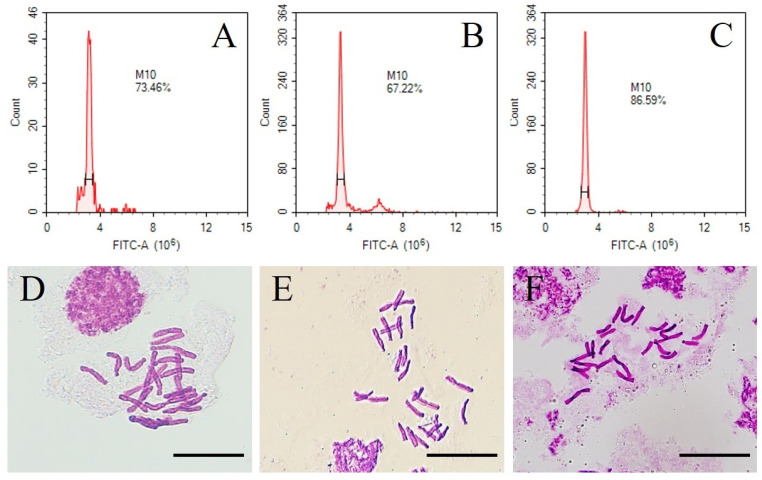
Chromosome ploidy in the LC×SQ-01 hybrid. (**A**–**C**) The chromosome ploidy in the hybrid and parents determined by flow cytometry; (**A**) is *L. callosum*, (**B**) is LC×SQ-01, and (**C**) is ‘Snow Queen’. (**D**–**F**) The chromosome number in the hybrid and parents determined via the root tip squashing method; (**D**) is *L. callosum*, (**E**) is LC×SQ-01, (**F**) is ‘Snow Queen’, and the bars represent 20 μm.

The female parent *L. callosum* and male parent ‘Snow Queen’ are diploid (2n = 2x = 24) plants. To elucidate the chromosome ploidy of the F1 hybrids, fresh leaves of the LC×SQ-01 hybrids and parents were examined via flow cytometry. The results (Figure 6A–C) revealed a similar ploidy level between the LC×SQ-01 hybrid and the parents. In addition, the chromosome number of the hybrid was consistent with that in the parents after squashing the root tip cells (Figure 6D–F). Therefore, the evidence indicated that LC×SQ-01 was a diploid plant, which corresponded to the general pattern of hybridization in diploid plants.

### 3.6. Detection of Antioxidant Enzyme Activities and the Botrytis Stress Response in the Hybrid

**Figure 7:**
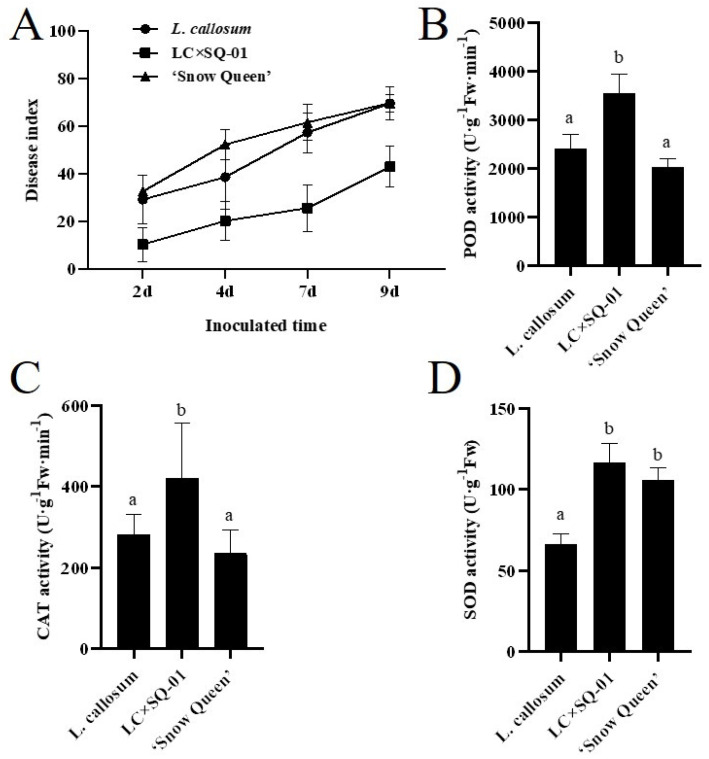
Comparisons of *Botrytis* stress response and antioxidant enzyme activity in the LC×SQ-01 hybrid and parents. (**A**) The incidence of the hybrid and the parents after inoculation with *B. cinerea* for different durations (2 dpi, 4 dpi, 7 dpi, and 9 dpi). (**B**–**D**) Analysis of peroxidase activity (POD), catalase activity (CAT), and superoxide dismutase activity (SOD) in the LC×SQ-01 hybrid and the parents *L. callosum* and ‘Snow Queen’, respectively. Different letters mean significant difference (*p* < 0.05).

The POD, CAT, and SOD enzyme activities of 60 d old plants were determined. The results showed that the activities of the antioxidant enzymes POD and CAT in the LC×SQ-01 hybrid were greater than those in the parents, while the SOD activity was greater only than that in the female parent *L. callosum* but was not significantly different from that in the ‘Snow Queen’ (Figure 7B–D). After 2 d, 4 d, 7 d, and 9 d of *Botrytis* inoculation, the pathogenesis of the LC×SQ-01 hybrid and parents was observed. The results showed that as the infection time increased, the incidence of *B. cinerea* in the LC×SQ-01 hybrid and parents became increasingly severe. In the early stage, the *Botrytis* response of the male parent ‘Snow Queen’ was more sensitive than that of the female parent *L. callosum* and the LC×SQ-01 hybrid (Figure 7A). However, the condition indices of the parents were comparable at 9 dpi (Table 5, Figure S2). In general, the LC×SQ-01 hybrid was moderately resistant, while the female and male parents were moderately susceptible, indicating heterosis. Overall, the increased *B. cinerea* resistance in the hybrid may be related to the greater antioxidant enzyme activity.

## 4. Discussion

Distant hybridization is an important means for breeding new lily germplasms. In recent years, significant progress has been made in lily breeding in China, and several new high-quality lily varieties have been bred. Using China’s endemic wild lilies as breeding materials and cross-breeding them with existing lily varieties is an effective way of selecting and breeding excellent lily varieties [7], and through such a method, several lily varieties with excellent agronomic traits have been bred, such as ‘Xingfushu’ [29], ‘Dandie’ [4], and ‘Longyahong’ [30]. Pan et al. (2018) [31] used six wild lilies (*Lilium lancifolium* Ker Gawl., *L*. *amoenum* E. H. Wilson ex Sealy, *L*. *henryi* Baker, *L*. *davidii* var. *willmottiae* (E. H. Wilson) Raffill, *L*. *rosthornii* Diels, and *L*. *taliense* Franch.) with five cultivated varieties (‘Siberea’, ‘Sorbonne’, ‘Viviana’, ‘Conca d’Or’, and *Lilium* ‘Robina’) to preliminarily obtain seven progeny strains by distant hybridization. This study attempted to carry out cross-breeding by using *L. callosum* as the female parent and cut lily flowers from ‘Snow Queen’ as the male parent to obtain high-quality hybrid offspring with high levels of resistance.

The incompatibility of distant hybrids of lily is mainly controlled by the style, and cut-style pollination can remove the obstacle of incompatibility; therefore, this method is expected to result in excellent hybrid offspring [23,24]. In this study, the cut-style method was used for crossing, and one enlarged fruit and a few seeds with embryos were obtained, which might be related to the pollen viability of the male parent ‘Snow Queen’ and the high-temperature environment after pollination. Previous studies have reported that the viability of ‘Snow Queen’ pollen is 54.5% [12], which is greater than the viability of the pollen used in this study (24.35%). Moreover, in summer, the temperature at the experimental site exceeded 30 °C on some days, and high temperatures not only inhibited pollen viability but also had a great impact on cross-breeding and fruiting [32].

The traits of hybrid progenies usually show extensive separability, and the traits often fall between the parents or closer to one of the parents [16,33,34]. The observations of the morphological and leaf epidermal characteristics of the open-field seedlings and tissue-cultured seedlings revealed that the seedling height, leaf size, and leaf width of the open-field seedlings of the LC×SQ-01 hybrid were between those of the parents; the length of the epidermal cells and the size of the guard cells of the tissue-cultured seedlings were between those of the parents; the width of the epidermal cells of the tissue-cultured seedlings was closer to that of the female parent *L. callosum*; and the shape and vertical wall shape were closer to those of the male parent ‘Snow Queen’. The conclusions drawn after morphological identification in this study were basically the same as those in previous studies. Characteristics, such as stomatal density and size, are often used to determine genetic relationships and are indirect indicators of the ploidy level of chromosomes [35]. Here, the stomatal density and stomatal size of the tissue-cultured seedlings of the LC×SQ-01 hybrid were between those of the parents, while the stomatal size of the open-field seedlings was closer to that of the female parent, which may be related to the light environment of the sampled leaves. In addition, the stomatal density of the open-field-grown seedlings was generally lower than that of the tissue-cultured seedlings, possibly because the CO_2_ concentration in the culture room with light was greater than that in the outdoor environment [36]. However, *L. callosum* was an exception, i.e., the stomatal density of the open-field seedlings increased instead of decreased. A possible reason for this finding is that the leaves of *L. callosum* are narrower, and a greater number of stomata facilitates adapt to changes in the climatic environment, indicating a strong self-regulation ability. The shapes of stomata can be roughly divided into three types: oval, wide oval, and oblong oval [19,20]. In this study, the stomata of both the open-field and tissue-cultured hybrid seedlings were oblong ovals, which is completely different from the shapes of the stomata of the parents.

Compared with morphological identification, the molecular identification of the hybrid progeny is more reliable. SSR markers are a molecular marker technology that is based on a specific primer PCR; this approach has the advantages of high polymorphism, good reproducibility, speed, and accuracy and has been applied in the identification of hybrids of different plants [37,38]. The hybridization process is usually performed strictly with bagging; therefore, the authenticity of the hybrids can be demonstrated when the hybrids and male parents have common bands [22]. In this study, five pairs of screened primers were used for hybrid identification, and among four pairs of SSR primers, male bands were amplified and found in the LC×SQ-01 hybrid, demonstrating the validity of the hybrid. In addition, for only one pair of SSR primers, the female bands were only amplified from the LC×SQ-01 hybrid. The possible reason for this finding is that the lily genome is highly heterozygous, and during meiosis, the DNA region of the marker locus could be altered due to the exchange of homologous chromosomes, resulting in the loss of the marker bands of the male parent in the offspring or the loss of a locus due to chromosome exchanges or base modification mutations of the DNA molecule in the process of gamete formation [39,40].

Studies on lilies have shown that if both female and male parents are normal diploid plants, the offspring of hybrids are usually diploids [41]. Both *L. callosum* and the ‘Snow Queen’ are diploid plants, and the LC×SQ-01 hybrid was determined to be diploid in this study, which is consistent with the conclusions of previous studies. Antioxidant enzymes, such as POD, CAT, and SOD, can scavenge excessive amounts of free radicals and reactive oxygen species (ROS) in plants and play important roles in plant stress adaptation. This study revealed that, in the hybrid, the activity levels of the antioxidant enzymes POD and CAT were greater than those in the parents, indicating that the LC×SQ-01 hybrid has greater environmental adaptability. Analysis of the *Botrytis* stress response of *L. callosum*, ‘Snow Queen’ and the hybrid showed that LC×SQ-01 had better resistance to *B. cinerea* infection than did the parents, which is consistent with the findings of Liu et al. (2019) [42] and Li et al. (2020) [43] that the activities of antioxidant enzymes can affect resistance against *B. cinerea* infection in lily. These results provide a basis for the subsequent selection of new germplasms of cut lily flowers with high *Botrytis* resistance.

## 5. Conclusions

Through the cut-style pollination method, seedlings of an interspecific hybrid progeny of *L. callosum* × ‘Snow Queen’ were obtained. The observations of the tissue-cultured and open-field seedlings revealed that the characteristics of the LC×SQ-01 hybrid, such as height, leaf morphology, epidermal cell, and stomatal density and size, mostly fell between those of the two parents, while some characteristics were closer to those of one of the parents. A total of 14 pairs of polymorphic SSR primers were amplified from the parents, and LC×SQ-01 was validated via the use of five pairs of those primers. Flow cytometry and chromosome counting demonstrated that the LC×SQ-01 hybrid was a diploid plant, which is consistent with the ploidy level of the parents. In addition, the activities of POD and CAT in the hybrid were much greater than those in the parents, and the resistance to *B. cinerea* infection was greater than that in the parents, revealing a certain degree of heterosis. This study provides an important basis for the breeding of ornamental lily varieties with an excellent flower colour and relatively strong resistance.

## Figures and Tables

**Table 1:** The results of crosses between *L. callosum* and the ‘Snow Queen’.

Cultivation Methods	Pollinated Flower Numbers	Number of Fruit Set	Fruit Set Rate	Number of Embryo Seeds	Embryo Seed Rate (%)	Number of Seedlings	Seedling Rate (%)
Ovary culture	35	0	0	—	0	0	0
Field culture	33	4	12	26	1.4	7	26.9

**Table 2:** Morphological characteristics of the hybrid and parental seedlings.

Types	Parent and Hybrid	Seedling Height (cm)	Leaf Length (cm)	Leaf Width (cm)	Rooting Characters
A	*L. callosum*	6.60 ± 0.39 a	4.91 ± 0.29 a	0.12 ± 0.01 c	Many, thicker, and shorter
	LC×SQ-01	4.45 ± 0.52 b	3.71 ± 0.35 b	0.16 ± 0.01 b	Many, thicker, and shorter
	‘Snow Queen’	2.45 ± 0.18 c	2.39 ± 0.19 c	0.33 ± 0.04 a	Fewer, thinner, and shorter
B	*L. callosum*	8.09 ± 0.56 a	6.08 ± 0.26 a	0.18 ± 0.01 c	Fewer, thinner, and longer
	LC×SQ-01	6.59 ± 0.39 b	5.08 ± 0.41 b	0.27 ± 0.02 b	More, thicker, and longer
	‘Snow Queen’	6.70 ± 0.54 b	5.25 ± 0.45 b	0.62 ± 0.05 a	Many, thicker, and longer

Note: A represents tissue-cultured seedlings, B represents open-field seedlings. Different letters behind each column mean significant difference (*p* < 0.05).

**Table 3:** The features of leaf epidermis cells in the hybrid and the parents.

Types	Parent and Hybrid	Epidermis	Cell Length (μm)	Cell Width (μm)	Shape of Epidermis Cell (μm)	Anticlinal Wall Pattern
A	*L. callosum*	Lower	683.89 ± 31.57 a	32.11 ± 1.56 b	Long bars	Shallow wave
		Upper	520.63 ± 25.08 a	46.70 ± 1.10 b	Irregular shape	Deep wave
	LC×SQ-01	Lower	502.16 ± 26.29 b	33.66 ± 1.06 b	Irregular shape	Shallow wave
		Upper	397.41 ± 14.93 b	50.81 ± 1.66 b	polygon	straight
	‘Snow Queen’	Lower	295.73 ± 19.76 c	41.55 ± 0.83 a	Irregular shape	Shallow wave
		Upper	254.03 ± 8.71 c	69.17 ± 1.90 a	polygon	Shallow wave
B	*L. callosum*	Lower	699.08 ± 38.44 a	31.92 ± 1.09 a	Long bars	straight
		Upper	809.11 ± 28.38 a	44.94 ± 1.13 c	Long bars	Shallow wave
	LC×SQ-01	Lower	521.05 ± 25.24 b	34.34 ± 1.45 a	Irregular shape	Deep wave
		Upper	473.06 ± 24.51 b	62.76 ± 2.78 b	Irregular shape	Deep wave
	‘Snow Queen’	Lower	335.41 ± 15.84 c	35.33 ± 1.72 a	Irregular shape	Deep wave
		Upper	354.55 ± 12.76 c	85.99 ± 3.11 a	polygon	Shallow wave

Note: A represents tissue-cultured seedlings, B represents open-field seedlings. Different letters behind each column mean significant difference (*p* < 0.05).

**Table 4:** The features of the stomal apparatuses in the hybrid and the parents.

Types	Parent and Hybrid	Stoma Density (mm^2^)	Stoma Length (μm)	Stoma Width (μm)	Guard Cell Length (μm)	Guard Cell Width (μm)	Shape of Stoma Apparatus
A	*L. callosum*	36.92 ± 2.03 b	28.27 ± 1.59 b	20.09 ± 2.60 b	68.81 ± 1.29 b	18.61 ± 0.51 b	Oval
	LC×SQ-01	52.33 ± 3.87 a	42.35 ± 1.27 a	18.36 ± 0.67 b	78.32 ± 1.51 a	19.09 ± 0.31 b	Long oval
	‘Snow Queen’	54.42 ± 2.93 a	44.09 ± 1.40 a	26.30 ± 0.75 a	81.90 ± 2.07 a	21.30 ± 0.58 a	Wide oval
B	*L. callosum*	40.78 ± 3.44 a	39.07 ± 0.71 b	14.37 ± 0.48 c	65.36 ± 1.09 c	16.24 ± 0.57 c	Oval
	LC×SQ-01	39.01 ± 4.83 a	45.16 ± 1.61 a	14.07 ± 0.23 b	78.73 ± 1.67 b	18.76 ± 0.41 b	Long oval
	‘Snow Queen’	28.57 ± 2.07 b	47.84 ± 0.62 a	22.80 ± 0.41 a	82.76 ± 0.87 a	20.22 ± 0.34 a	Wide oval

Note: A represents tissue-cultured seedlings, B represents open-field seedlings. Different letters behind each column mean significant difference (*p* < 0.05).

**Table 5:** In vitro analysis of the susceptibility of the hybrid to *B. cinerea* at 9 dpi.

Varieties	Incidence (%)	Disease Index	Relative Disease Resistance Index	Anti-Inductive Types
*L. callosum*	92.98	69.62	0.30	Medium sensitivity
LC×SQ-01	69.28	42.95	0.57	Medium resistance
‘Snow Queen’	98.33	69.82	0.30	Medium sensitivity

## Data Availability

All the supporting data can be found as an additional file along with this manuscript. The materials are available from the corresponding authors upon reasonable request.

## Supplementary Materials

The following supporting information can be downloaded at: https://www.mdpi.com/article/10.3390/plants13101376/s1, Table S1: Primers used for screening polymorphic SSR marker primers. Table S2: SSR primers for amplifying polymorphic bands from the two parents. Figure S1: Morphological features of tissue-cultured and open-field-generated plants. (A–C) Representative seedlings of *L. callosum*, LC×SQ-01 and ‘Snow Queen’, respectively, cultured for 60 days in medium; the bars represent 1 cm. (D–F) Representative seedlings of *L. callosum*, LC×SQ-01 and ‘Snow Queen’, respectively, grown for 60 days in open fields; the bars represent 1 cm. Figure S2: Comparisons of *Botrytis* stress response in the scales of the LC×SQ-01 hybrid and the parents. A, B, C: Representative images of the scales from *L. callosum*, LC×SQ-01 and ‘Snow Queen’ after inoculation with *B. cinerea* at 9 days (9 dpi), respectively. The bars represent 1 cm (A–C).
