# Supplementary material for: Obtaining and Characterization of an Interspecific Hybrid between Lilium callosum and ‘Snow Queen’ and Evaluation of the Botrytis Stress Response
Source: Plants (Basel). 2024 May 15;13(10):1376. doi: 10.3390/plants13101376 (PMC11125225; doi:10.3390/plants13101376)
Supplement: Supplementary file 1 [file plants-13-01376-s001.zip › Supplementary Figures.pdf]

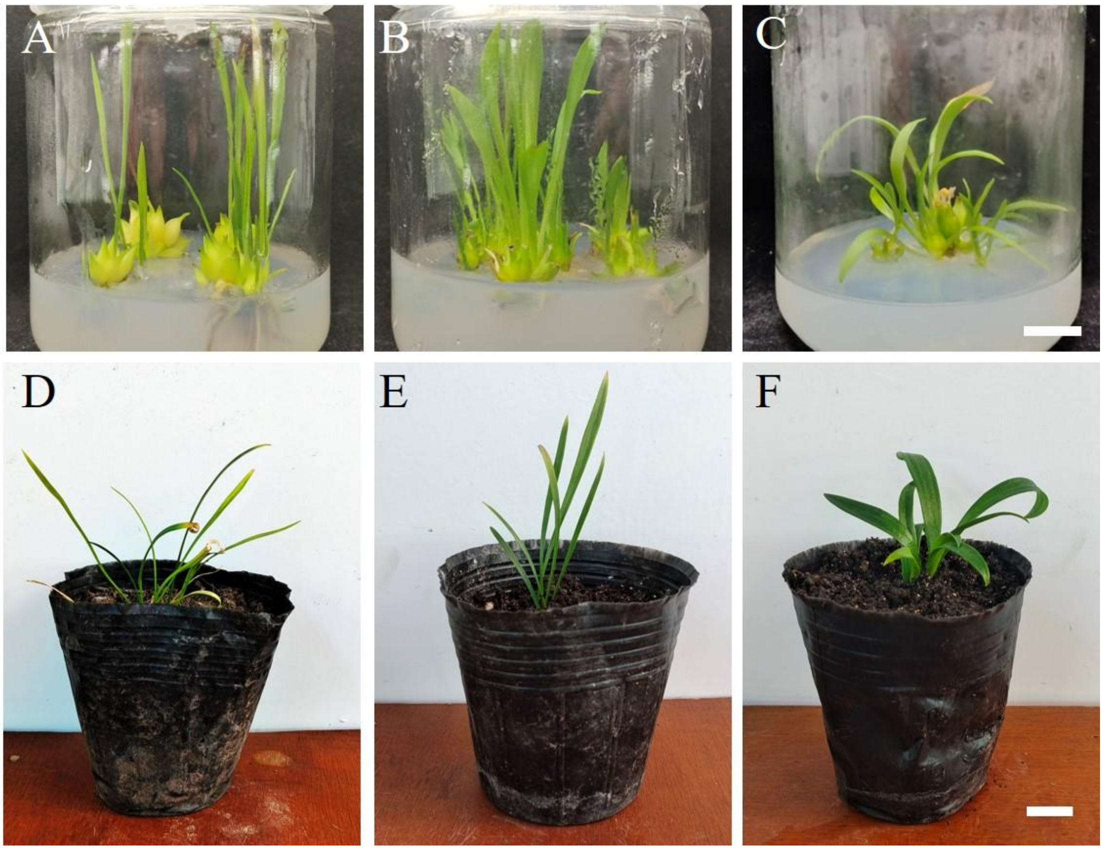

Figure S1. Morphological features of tissue-cultured and open-field-generated plants. A, B, C: Representative seedlings of *L. callosum,* LC×SQ-01 and 'Snow Queen', respectively, cultured for 60 days in medium; the bars represent 1 cm. D, E, F: Representative seedlings of *L. callosum,* LC×SQ-01 and 'Snow Queen', respectively, grown for 60 days in open fields; the bars represent 1 cm.

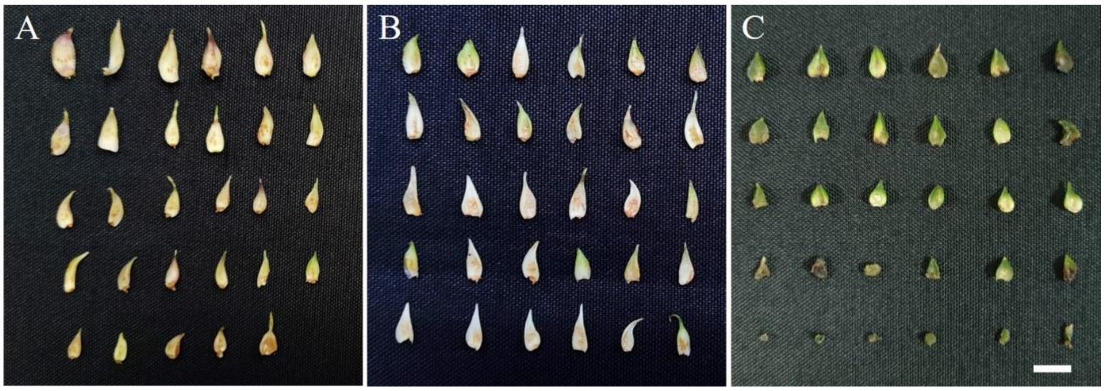

Figure S2. Comparisons of *Botrytis* stress response in the scales of the LC×SQ-01 hybrid and the parents. A, B, C: Representative images of the scales from *L. callosum*, LC×SQ-01 and 'Snow Queen' after inoculation with *B. cinerea* at 9 days (9 dpi), respectively. The bars represent 1 cm (A-C).
