# Supplementary material for: Obtaining and Characterization of an Interspecific Hybrid between Lilium callosum and ‘Snow Queen’ and Evaluation of the Botrytis Stress Response
Source: Plants (Basel). 2024 May 15;13(10):1376. doi: 10.3390/plants13101376 (PMC11125225; doi:10.3390/plants13101376)
Supplement: Supplementary file 1 [file plants-13-01376-s001.zip › Supplementary Tables.pdf]

Table S1. Primers used for screening polymorphic SSR marker primers.

No.	Name	Sequences（5'— 3'）	Name	Sequences（5'— 3'）
1	ivflmre17-F	GTAGGCGGGCTTTCCTTG	ivflmre17-R	TGGCCTCCTGCTTACCTT
2	ivflmre18-F	GTCTCCCGCCCCGCCCT	ivflmre18-R	CCCCCACACTATCCACAG
3	ivflmre19-F	CGTTGTCGCGGCTGCAG	ivflmre19-R	CCACCGCAACGCCACTG
4	ivflmre21-F	TCTTCAGTTAGAGGCTTAGG	ivflmre21-R	CTAATAGGCAAGATTTACGAC
5	ivflmre26-F	GATGGGGCAGGCATGGTG	ivflmre26-R	AGTGAGGAGGACGGGGAGG
6	ivflmre28-R	TTGATTGCATTTTTGGGTCTC	ivflmre28-R	TTGATTGCATTTTTGGGTCTC
7	ivflmre30-F	CATCTCCGGCCAAATTGCAT	ivflmre30-R	CCCCCACCGCCGAACAAT
8	ivflmre67-F	GCTGGTCGCCATTTTTTAG	ivflmre67-R	CTCTCCCTTCACCTTCTCG
9	ivflmre73-F	TCTAAATAAGAAGCCCACCA	ivflmre73-R	GGAATCACGAGAGATACCCA
10	ivflmre107-F	TGTAACCCTTGACATAACCAT	ivflmre107-R	ATAGCCCTATATCCTGTCACT
11	ivflmre108-R	ACCGGTCGGTCGAGAAGAT	ivflmre108-R	ACCGGTCGGTCGAGAAGAT
12	ivflmre110-F	TTCTCTCTCATGGCCTCCTATT	ivflmre110-R	ACTTTGCATCGATGGTTACC
13	ivflmre112-F	TAGGGTTTTTCTGTCTCTCC	ivflmre112-R	AACTGTCTCCATATGCGTCG
14	ivflmre114-F	AACTACCACTCTTCATCGCTC	ivflmre114-R	GGCTTCTCTTTTTTATTCTGC
15	ivflmre116-F	CTCGGTGTAAATCCTGACGC	ivflmre116-R	TCCCCCCTCTTATGAGTACG
16	ivflmre117-F	CATCCCACACACCAACACCT	ivflmre117-R	TCCACGACCACCGACTAGC
17	ivflmre123-F	GTCGGTGATGGACGGTTCT	ivflmre123-R	TTGCTCGGATACATGAAAAC
18	ivflmre124-F	GCCTCAGATCCGTTCTACAT	ivflmre124-R	TCTCCACCATAAGGGTTCTG
19	ivflmre173-F	CGGCTTCTTTCACTCCTATCTC	ivflmre173-R	CCAAACCCCAGGAAAGAGG
20	ivflmre176-F	AGATCGGGGGAGCATGTCAG	ivflmre176-R	GGCGGAGGTAGAGATCGAGG
21	ivflmre177-F	GGTCGTTTCTCTTCATTGTT	ivflmre177-R	CAAAGATGGAAATAAACTCG
22	ivflmre178-F	CAACAATGGCATCACCAAAT	ivflmre178-R	GGAGGATGGAGTTGAAGACG
23	ivflmre180-F	CACCGCAACGCCGAGCAG	ivflmre180-R	GAAGGTGAGGACGGCGAGGG

24	ivflmre181-F	GGAGGATGGATGCCGATACC	ivflmre181-R	CCCACATCGTATCCGAGAAGA
25	ivflmre182-F	CCTGGGTGCAGTCTTGGGAG	ivflmre182-R	CGCCATTGCCTTAGGGTTTC
26	ivflmre183-F	TCATCCTCCACCGCTGTCTC	ivflmre183-R	ATTGGGGGTGGAAACTGAGG
27	ivflmre189-F	CATTTAATCCTCGTTATTCACT	ivflmre189-R	GTGTAAAAGCAACATGGTCG
28	ivflmre193-F	GTTGCAGGTTGTGAGGTGTT	ivflmre193-R	ATGTTTTCGTATGCACCTTG
29	ivflmre197-F	GGGTCCGACTCTCCCTCTTC	ivflmre197-R	ACACCACAAACCCAACCCTAC
30	ivflmre203-F	CTCCACAGAGGTCCCCACAA	ivflmre203-R	GTTCCCAACGCACCCCACAC
31	ivflmre205-F	CTCTATTTCCCTCCCGGTCT	ivflmre205-R	GCTGAGATGAGCCAGACAAG
32	ivflmre286-F	ACCAGTATCACCTTGGCACC	ivflmre286-R	CCAAAGCAGGCACAGAAACT
33	ivflmre287-F	TGCATCAAATCGCACATGTTAG	ivflmre287-R	TGGACCCACTTGCCATCTTC
34	ivflmre295-F	GCACCATCTGCGGAAACCTA	ivflmre295-R	ACCGGCAGCGACTTGGACT
35	ivflmre303-F	GATGTAATATCTTAATGCCTGTCG	ivflmre303-R	ACTTGGCGGTGGTCACGG
36	ivflmre304-F	GATGGAAAGCATTCACGGCA	ivflmre304-R	CTCATTCCACAATAAACTGATCTT
37	ivflmre305-F	CCTCGGCATCGGAGAACTCA	ivflmre305-R	GAAGCGGCTGGTGAAGAAGA
38	ivflmre310-F	GCGGAGATGGTGTGATTTTC	ivflmre310-R	TCCTCCTCTCTAGTCCTGCTC
39	ivflmre314-F	TTTCTCCCCTGCTGAAGTGG	ivflmre314-R	CCAACAACGAGCAACCCATC
40	ivflmre315-F	TGAGCCGTAATCGGAAGCC	ivflmre315-R	ACAACCCTCAGAGACTTCAATG
41	ivflmre318-F	CAGTTTTTATTGGCAACAGAT	ivflmre318-R	GTTAATGACTCAACGGCAGA
42	ivflmre551-F	CACCCAAATCAGCCAAATCC	ivflmre551-R	TGTCATTGCTCCAAGAGAAGG
43	ivflmre558-F	CTCATTGTCCGAAAGCTC	ivflmre558-R	CCAGTCATTCCTCTTCCT
44	ivflmre560-F	GATGTAGAGTCGGGGGCT	ivflmre560-R	CAAGAGTCCCCACACCATC
45	ivflmre563-F	CTCCATTCTCTCCAAACTCTC	ivflmre563-R	CAAATATCAGCATGAACCAGC
46	ivflmre570-F	TCTCCTTCTCCAACCATGA	ivflmre570-R	AATTCTCGTCCAGCAAAAG
47	ivflmre576-F	ATGGTGCCGATGTTGACG	ivflmre576-R	CCAAACCCTATCTCATCCAAT
48	ivflmre577-F	TTCCTTCGCATGTCCACC	ivflmre577-R	AATCTCAGCTATTCACCACAG

49	ivflmre578-F	CAAATATGTGTCGAACAGATG	ivflmre578-R	CCTTCCCTTCTCCCTTTCT
50	ivflmre580-F	GATGGGCGGTTGCTTCTA	ivflmre580-R	AAGCCGTGGAGGAAGAGG
51	ivflmre582-F	CCGGCAATAGCTGAGATC	ivflmre582-R	GTTGGTCCCGTTGATTCT
52	ivflmre583-F	AAAAGCAGAGCAGAGAATTG	ivflmre583-R	CGAGACGAAGAGCAACCAC
53	ivflmre590-F	TCGCTTCTCCTTTCTTTGAT	ivflmre590-R	GGTGCGGAAGGTTGTGAG
54	ivflmre676-F	CTCTCGCCCAGGGACCAC	ivflmre676-R	CCACGGAACCGCCAGATA
55	ivflmre720-F	GAAGAAGTAACATGGTGGTCA	ivflmre720-R	CATAGCAACTGCGGTAAGAG
56	ivflmre724-F	ACCCATTTGGAAGTGCCT	ivflmre724-R	CTGGTTCGCTGATTCTCG
57	ivflmre726-F	GGGTCTCAGTCAAGCACA	ivflmre726-R	GCGGCATCTATAAATCGTC
58	ivflmre730-F	TGTTAGCAGCGGCATCGG	ivflmre730-R	ACTCTTCGGCACGGCATT
59	ivflmre732-F	GCAGACCTCAAGCGATAA	ivflmre732-R	GAACCCCTGGAGCTGATC
60	ivflmre733-F	GGCCCTCTTTGTTGTTCT	ivflmre733-R	CTGTCCTCCGCTTCCATT
61	ivflmre736-F	TCCACCCACTTACCACCT	ivflmre736-R	TGAGACTGTTTCAGGCATC
62	ivflmre739-F	GTCCGCATAATACCAACC	ivflmre739-R	TTGATGATAACGAAAGAGGA
63	ivflmre740-F	CTACTCTGCTGTCCTCCGTG	ivflmre740-R	CAAAAGTCCAGGCAAATCA
64	ivflmre743-F	TAGATGTGAATGTGCAGACTT	ivflmre743-R	CAATTGACTGCGATGCTACT
65	ivflmre745-F	CAGTTCAACGGACGTGGA	ivflmre745-R	GCTGATAATCGGTGAACTCC
66	ivflmre747-F	CAGATTTCTTGTCAGCCCTA	ivflmre747-R	CAGTTCTACATCATTCCCTTC
67	ivflmre748-F	ACTTCTGCCATCTGAGCCT	ivflmre748-R	CGCCTCTCATGAGCACGA
68	ivflmre752-F	GCTTTGCTGAGGCATCGT	ivflmre752-R	TGGTCCCAAACGGGTATT
69	ivflmre755-F	CTCTGCATCTTAGGTTGGC	ivflmre755-R	AAGAGGGCATAAACGGAAT
70	ivflmre758-F	AAAACCCTGGGCACTGGA	ivflmre758-R	TTGGACGGCATAACCTTG
71	ivflmre759-F	CGCCGATTCAAAAATTACA	ivflmre759-R	GCCGTCTTGCTTGCCTAT
72	ivflmre761-F	GGGAGTTACCTTAAGATGTCA	ivflmre761-R	GCTTCAGTCCCTCCCAGTA
73	ivflmre763-F	GAGCCCGAAATTCCAGAT	ivflmre763-R	TCCTTCCGCTAACTCAAT

74	ivflmre765-F	CGCGTCGTCGTTCTGGTC	ivflmre765-R	AAATCTTCCTTGGCATCCG
75	ivflmre769-F	GTCTTATCACGTGGGCCG	ivflmre769-R	GCAAGAACCTGATTTGATGAA
76	ivflmre774-F	TGAAGATGGTAAACCAAGCG	ivflmre774-R	CATGGTTTCTCGGCCTCC
77	ivflmre777-F	ACCCCTACCGAAGTTCCC	ivflmre777-R	CAAGGCGGTACTTCTGTTC
78	ivflmre787-F	CCTAAGAAAGGACCACCAAT	ivflmre787-R	GTAAGGGATCAAACACTAAGC
79	ivflmre789-F	TTTCCCAGGTGGCAGAGC	ivflmre789-R	TGCCGCGTTCAACCTCTA
80	ivflmre790-F	GGTAAAACTGGGTGAACTCG	ivflmre790-R	AGAGCCCAGAGCTATCACAG
81	ivflmre791-F	GGGCTTCTCGAATCCGTAC	ivflmre791-R	ATCCTGGTGGGTCTCCGG
82	ivflmre792-F	GATAGGAGTCTGAGGCTGTG	ivflmre792-R	TTGTGACTTGGTAGCGGC
83	ivflmre793-F	AACCTAATCCTACTCAAGCTG	ivflmre793-R	TCTGACACTGCCGAGGTAT
84	ivflmre794-F	CTGCTGAGCGAGGAAATG	ivflmre794-R	GTAAAAGCATGGTTCTTGAGGT
85	ivflmre795-F	TGCTCCGTACTCCACTATCT	ivflmre795-R	CGCCGAAGCATAATCACT
86	ivflmre796-F	AGTCCCAGATCCAATTCGA	ivflmre796-R	GGTGACCGGGGACGTATT
87	ivflmre800-F	AGAATTACCTGATCTGCGC	ivflmre800-R	TCATCCAATCTTTGCCATAT
88	ivflmre802-F	ACCTCCGATTGCCATATAC	ivflmre802-R	GAACCTGTCGCTTCATACTC
89	ivflmre839-F	CGGACTCAGACCACAGCT	ivflmre839-R	TTCGGTTCTCGGCTTGTT
90	ivflmre841-F	ATCAGAGTACATCCCACAGC	ivflmre841-R	ATTGTGACTAAATCCGACTAAC
91	ivflmre844-F	CAACTTCATTGTCGGCATC	ivflmre844-R	AGAAGCCGTATCCCGACT
92	ivflmre879-F	TCCAGAACAGGAGGTATGC	ivflmre879-R	AGCCTGTAAACCGAACCC
93	ivflmre885-F	TGAACTAAATCTCGTCCTGC	ivflmre885-R	GATCTTGATCAGAAACAGGAG
94	ivflmre886-F	CACAGTGACAAGAGGAACATT	ivflmre886-R	TGATTGTTTCGCAGGTTATG
95	ivflmre890-F	CAGGTCTGTCCTCGTTCC	ivflmre890-R	CCACAGCCCTAAGATTCAC
96	ivflmre894-F	AGGCATCATAGGAGGTGG	ivflmre894-R	TCCGTCCAAGGAAAGTGA
97	ivflmre895-F	AATGCACAACGCACTCAA	ivflmre895-R	AGAGTGTGAAGGTTATGCATC
98	ivflmre897-F	AAGGCAGACTTCCGACAT	ivflmre897-R	CCACCAAGCCATTCATTC

Table S2. SSR primers for amplifying polymorphic bands from the two parents.

No.	Name	Female parent[#]	Male parent[*]
1	ivflmre18	√	—
**2**	**ivflmre19**	√	√
3	ivflmre30	—	√
**4**	**ivflmre67**	√	√
**5**	**ivflmre108**	√	√
6	ivflmre117	—	√
7	ivflmre173	—	√
8	ivflmre180	—	√
**9**	**ivflmre181**	√	√
**10**	**ivflmre197**	√	√
11	ivflmre203	—	√
12	ivflmre286	—	√
**13**	**ivflmre303**	√	√
**14**	**ivflmre304**	√	√
15	ivflmre318	—	√
**16**	**ivflmre560**	√	√
**17**	**ivflmre563**	√	√
**18**	**ivflmre580**	√	√
19	ivflmre583	√	—
20	ivflmre590	—	√
21	ivflmre724	√	—
22	ivflmre732	√	—
**23**	**ivflmre740**	√	√
**24**	**ivflmre745**	√	√
25	ivflmre748	—	√
26	ivflmre752	√	—
27	ivflmre765	√	—
28	ivflmre787	—	√
29	ivflmre790	√	—
**30**	**ivflmre791**	√	√
31	ivflmre792	—	√
32	ivflmre839	√	—
**33**	**ivflmre844**	√	√
34	ivflmre879	√	—

Note: the bold means primers amplifying polymorphic bands from the two parents, # means primers amplifying polymorphic bands from the female parent, * means primers amplifying polymorphic bands from the male parent.
